# Eight-Week Resistance Training and Manual Therapy in Young Patients with Severe Hemophilia: A Case Series Evaluating Functional, Imaging, and Immunological Outcomes

**DOI:** 10.3390/jcm14238419

**Published:** 2025-11-27

**Authors:** Krystian Guzmann, Bartosz Wilczyński, Marta Jaskulak, Julia Radoń-Proskura, Arkadiusz Szarmach, Andrzej Mital, Katarzyna Zorena

**Affiliations:** 1 Department of Immunobiology and Environment Microbiology, Faculty of Health Sciences, Medical University of Gdansk, 80-211 Gdansk, Poland; 2 Department of Paediatrics, Haemathology and Oncology, Medical University of Gdansk, 80-211 Gdansk, Poland; 3 2nd Department of Radiology, Faculty of Health Sciences, Medical University of Gdansk, 80-211 Gdansk, Poland; 4 Department of Hematology and Transplantology, Medical University of Gdansk, 80-211 Gdansk, Poland

**Keywords:** adolescent, hemophilia A, hemophilia B, resistance training, manual therapy, interleukin-18, nerve growth factor, receptor for advanced glycation end products, chemokine CCL2, intercellular adhesion molecule-1

## Abstract

**Background**: Hemophilia A and B are hereditary bleeding disorders that result in recurrent joint and muscle hemorrhages, leading to hemophilic arthropathy, muscle atrophy, and disability. Recent evidence suggests that physiotherapeutic interventions, including resistance training and manual therapy, may mitigate these effects, although comprehensive studies remain limited. This case series aimed to describe the outcomes of an eight-week physiotherapy program combining progressive resistance training and manual therapy in four adolescent boys (aged 11–17 years) with severe hemophilia. **Methods**: The program targeted joint function, muscle strength, ultrasound findings, and pain, with additional exploratory evaluation of neuroinflammatory and endothelial biomarkers: interleukin-18 (IL-18), C-C motif chemokine ligand 2 (CCL2), soluble intercellular adhesion molecule-1 (ssICAM-1), β-nerve growth factor (β-NGF), and soluble receptor for advanced glycation end-products (sRAGE). **Results**: After the intervention, Hemophilia Joint Health Score (HJHS) total scores decreased by 35–62%, indicating functional improvement, while muscle strength increased across most joints. No progression of arthropathy was observed on ultrasound (HEAD-US). IL-18 and ssICAM-1 levels decreased on average by 42% and 29%, respectively, whereas β-NGF and sRAGE increased by 39% and 11%, suggesting potential anti-inflammatory and neuroprotective responses. **Conclusions**: These descriptive findings indicate that individualized physiotherapy may serve as a supportive component of hemophilia care, while biomarker monitoring provides exploratory insight into treatment-related physiological responses.

## 1. Introduction

Hemophilia A and B are hereditary bleeding disorders caused by deficiencies in coagulation factors VIII and IX, respectively. Recurrent hemarthroses in these patients lead to progressive musculoskeletal complications, including hemophilic arthropathy (HA), muscle atrophy, and restricted joint mobility. HA, characterized by chronic synovial inflammation, cartilage degradation, and subsequent subchondral bone damage, remains a major cause of disability in this population [1]. Traditional therapeutic approaches in HA were based on avoiding physical activity, especially resistance training, due to concerns about the risk of recurrent injuries and exacerbation of bleeding [2,3,4].

However, advances in pharmacotherapy, including regular factor replacement and novel non-factor prophylactic therapies, have substantially reduced bleeding risk, allowing physical activity to emerge as a form of “prophylactic physiotherapy” [3]. In recent years, a growing body of research has highlighted the potential of physiotherapy, including progressive resistance training and manual therapy to improve joint function, muscle strength and quality of life in patients with hemophilia [5,6,7,8,9,10]. Manual therapy encompasses hands-on techniques including joint mobilization, soft tissue manipulation, and stretching to reduce pain, improve mobility, and promote musculoskeletal function. Nevertheless, despite promising results, there remains a lack of comprehensive randomized studies to confirm the effectiveness and safety of this integrated physiotherapeutic approach [5].

Previous research has shown that structured exercise programs integrating strength, coordination, and endurance can safely improve function and slow joint deterioration in individuals with hemophilic arthropathy [6]. Subsequent case-based work confirmed the feasibility of progressive resistance training even in patients with inhibitors, provided adequate prophylaxis is maintained [7]. Evidence from resistance-training studies in the general population further supports that moderate training volume (12–20 weekly sets) is sufficient to enhance muscle strength and mass without excessive load [8]. These findings informed the design of our intervention, which combined moderate-volume resistance training with adjunct manual therapy to optimize joint mobility and stability in adolescents with severe hemophilia. Moreover, personalized rehabilitation, as indicated by Scaturro et al. (2021), is crucial in improving functional outcomes and quality of life, especially in the early stages of HA [9]. Research by Cuesta-Barriuso (2018) highlights the benefits of manual therapy and home exercises in improving range of motion and reducing pain in patients with HA [11]. Despite evidence supporting the effectiveness of moderate training volume (12–20 weekly sets), there is a lack of studies that tailor this training programs to the individual needs of hemophilia patients, considering their history of bleeding, the severity of arthropathy, and differences in exercise tolerance.

Joint imaging in hemophilia commonly utilizes the Hemophilia Early Arthropathy Detection with Ultrasound (HEAD-US) protocol [12]. This standardized scoring system assesses synovial hypertrophy (0–2), cartilage damage (0–4), and subchondral bone alterations (0–2) in the elbow, knee, and ankle joints, enabling early detection of arthropathic changes. Despite its diagnostic value, its use for monitoring exercise-induced adaptations remains limited [13].

However, there is a lack of comprehensive data on the effects of exercise on selected biochemical markers that may be associated with inflammatory processes in the joints. Chronic inflammation is a central mechanism driving HA pathogenesis [14,15]. Recurrent hemarthroses trigger iron deposition within the synovium, activating immune cells and inducing the release of pro-inflammatory cytokines and degradative enzymes. This cascade perpetuates synovitis, cartilage destruction, bone remodeling, and pain. Consequently, biomarkers reflecting these inflammatory pathways—such as interleukin-18 (IL-18), C-C motif chemokine ligand 2 (CCL2, also known as monocyte chemoattractant protein-1), intercellular adhesion molecule-1 (sICAM-1), β-nerve growth factor (β-NGF), and soluble receptor for advanced glycation end-products (sRAGE)—may provide insights into disease activity and therapeutic response [16,17,18]. Their quantification could enable early detection of subclinical inflammation and offer potential targets for novel interventions, including IL-18 inhibition, CCL2 neutralization, or modulation of RAGE pathways [17].

CCL2 is a potent chemokine that mediates monocyte, macrophage, and T-cell recruitment to inflamed synovium. In HA, elevated CCL2 promotes chronic synovitis, cytokine secretion (IL-1β, TNF-α), cartilage degradation, and bone remodeling [17]. In sickle cell anemia, high CCL2 levels correlate with pain severity and earlier onset of vaso-occlusive crises, suggesting its potential as a prognostic biomarker [16]. SsICAM-1, a cell adhesion molecule expressed on endothelial cells, leukocytes, and fibroblasts, is upregulated by pro-inflammatory cytokines (e.g., TNF-α, IL-1β). Elevated SsICAM-1 levels in other chronic inflammatory disorders—such as rheumatoid arthritis and atherosclerosis—correlate with disease activity and tissue damage, implying that in hemophilia, SsICAM-1 may serve as an early indicator of arthropathy exacerbation preceding imaging changes [16,17,18]. Another proinflammatory cytokine is interleukin 18 (IL-18), which plays an important role in both innate and adaptive immunity. IL-18, produced mainly by activated macrophages through NLRP3 inflammasome activation and caspase-1 processing, contributes to both innate and adaptive immune responses. In hemophilia and other chronic inflammatory conditions, IL-18 enhances Th1-type immunity, promotes interferon-γ and TNF-α production, recruits monocytes, and sensitizes nociceptors—linking inflammation to neuropathic pain [17].

Nerve growth factor beta (β-NGF) is a neurotrophin responsible for the development, survival and function of sensory, sympathetic and cholinergic neurons. In addition to its developmental functions, β-NGF plays a key role in neuroplasticity and pain modulation processes. In a study of patients with SCD, β-NGF levels were significantly higher in plasma compared to healthy subjects, interestingly, higher levels of β-NGF correlated negatively with pain severity, number of pain episodes and depression scale [16]. This suggests that in patients with SCD, NGF may have an adaptive function, potentially compensating for the effects of chronic pain and emotional stress. Rather than exacerbating nociceptor sensitization (as in other chronic diseases), it may have a neuroprotective or regenerative effect here [16]. In the context of hemophilia: β-NGF has been analyzed as a neuroinflammatory marker and potential indicator of joint pain and nervous system response to chronic inflammation and joint degeneration [18].

sRAGE is a soluble form of the Receptor for Advanced Glycation End-products (RAGE), which in its membrane form plays a role as a sensor of tissue damage and modulator of inflammation. Wehmeier’s study involving patients with hemophilia (PWH) found significantly higher levels of sRAGE compared to the control group; however, contrary to expectations, there was no significant correlation between sRAGE levels and clinical parameters such as pain intensity, number of bleeds or imaging findings [16]. sRAGE is a complex indicator of inflammatory balance that may have a protective effect in various clinical contexts. In hemophilia, high levels of sRAGE may be indicative of activation of compensatory systems, rather than directly indicative of symptom severity [16,17,18,19].

Given the limited availability of disease-modifying therapies for HA, identifying reliable biomarkers is essential for risk stratification and monitoring of therapeutic effects [15]. Therefore, the present case series describes the outcomes of an eight-week physiotherapy program combining progressive resistance training and manual therapy on functional, biochemical, and radiological outcomes in patients with severe hemophilia. Specifically, it reports changes in muscle strength, functional capacity, pain, imaging, and circulating biomarkers associated with hemophilic arthropathy.

## 2. Materials and Methods

### 2.1. Study Design

This study was designed as a prospective descriptive case series describing the functional, biochemical, and radiological outcomes of adolescents with severe hemophilia undergoing an eight-week physiotherapy program combining progressive resistance training and manual therapy. The study followed the principles of the CARE (CAse REport) guidelines for accurate and transparent reporting of clinical case series. Ethical approval was obtained from the Bioethics Committee of the Medical University of Gdańsk (NKBBN/425/2023), and written informed consent was obtained from all participants and their guardians in compliance with Declaration of Helsinki. Pre-registration with study protocol was done in Open Science Framework (https://doi.org/10.17605/OSF.IO/EUR6P).

### 2.2. Participants

Participants were recruited through their attending physician at the Department of Pediatrics, Hematology and Oncology at the University Clinical Center in Gdańsk in 2024–2025. Eligible participants included adolescents between the ages of 11 and 17 years. All participants had confirmed severe hemophilia, with factor VIII or IX activity levels below 1%, verified through their medical records. To minimize the risk of exacerbation of hemarthrosis during the intervention, only patients who had no new joint hemorrhages within two preceding weeks before the study were included.

All assessment of the patients was performed before physiotherapy program, after eight-weeks of training. Examination included HJHS of elbow, knee, and ankle joints, as well as ultrasonography (HEAD-US) scoring system was performed at the same time points by radiologist. Quality of life (QoL) questionnaires were answered before and after training program.

Each patient had regular prophylaxis of bleedings with a different hematologic medication, based on individual pharmacokinetics in cases 1, 3 and 4. Each physiotherapy session was scheduled on the same day as prophylactic factor administration, typically 2–3 h post-injection, to ensure optimal hemostatic coverage during exercise, in accordance with clinical guidelines. Annual bleeding rate (ABR) and annual joint bleeding rate (JABR) were recorded at the beginning of the study and reassessed 6 months after the physiotherapy program.

### 2.3. Case Presentation

#### 2.3.1. Case 1

We focused on a 17-year-old male patient with a severe hemophilia A with regular prophylactic recombinant factor VIII concentrate substitution (3 × 2000 IU intravenously per week). The patient was diagnosed with hemophilia at the age of 9 months and experienced recurrent intraarticular and muscle bleeds, though no internal or brain hemorrhages had occurred. His medical history included port-a-cath implantations and removals (2008, 2015) and self-administration of intravenous factor VIII into peripheral veins. A family history of hemophilia was confirmed, with the patient’s mother identified as asymptomatic carrier (factor VIII activity 112%). The patient was in good general condition and no active bleeding at the time of assessment. Additionally, he was diagnosed with Asperger syndrome.

Initial multiple joints ultrasound revealed moderate synovial hypertrophy in the dorsal aspect of the left radial-carpal joint, overload lesions on the extensor IV trochlea of the left hand, uneven articular cartilage in the right shoulder joint, left knee and both ankles, and a fracture gap in the posterior horn of the medial meniscus of the right knee, the HEAD-US score was 4. Laboratory tests, including full blood count and biochemistry were within the normal range, inhibitor of factor VIII was negative.

#### 2.3.2. Case 2

We focused on a 16-year-old male patient with severe hemophilia A, complicated by a high titre inhibitor of factor VIII (>200 Bethesda units/mL) and failure of immune tolerance induction therapy (ITI). The patient in the past had a history of HA affecting the knee and elbow joints, recurrent bleeding in target joints (left knee and left elbow), and prolonged urinary tract bleeding as well as severe bleeding after tooth extraction.

The patient was in good general condition, and there were no signs of active bleeding at the time of evaluation. Current bleeding prophylaxis included nonfactor treatment with emicizumab subcutaneously every two weeks, in case of bleeding patient would be treated with inhibitor bypassing agent such as recombinant activated factor VII (rVIIa) as a first choice.

Initial joint ultrasound showed increased synovial effusion and hypertrophy in both first metatarsophalangeal (MTP1) joints. Although no effusion or synovial hypertrophy in the ankle joints were revealed, uneven cartilage outline on the block of the left ankle and signs of previous anterior talofibular ligament (ATFL) injury in the same joint. Small fracture gap in the posterior horn of the medial meniscus of the right knee joint and grade II/III chondropathy in the left knee were detected. Normal articular cartilage patterns, smooth bony outlines, and normal ultrasonographic images of the hip, wrist, shoulder, and elbow joints. the HEAD-US score was 5. Basic laboratory tests were within normal ranges, inhibitor of factor VIII undetectable (<0.5 Bethesda units) though patient hasn’t received factor VIII concentrate for several years.

#### 2.3.3. Case 3

We focused on an 11-year-old male patient with severe hemophilia B and bleeding phenotype with increased demand for prophylactic regular administration of recombinant factor IX concentrate (3× 2750 IU intravenously per week). Inhibitor of factor IX was negative. Due to repeated bleedings into his left ankle (target joint), he underwent two procedures of radiosynovectomy with good final result.

The patient was in good general condition and no active bleeding.

Initial joint ultrasound revealed homogeneous effusion in the of the left knee joint (10 × 30 mm), fracture gap in the posterior horn of the medial meniscus of the left knee joint, and homogeneous effusion in the first metatarsophalangeal (MTP1) joint of the right foot (up to 3.2 mm). Normal articular cartilage pattern and smooth bony outlines. The HEAD-US score was 3. Basic laboratory tests were within normal limits.

#### 2.3.4. Case 4

We focused on a 15-year-old male patient with severe hemophilia A with HA, overweight, observed for hypertension. The patient was in good general condition, with efficient circulatory and respiratory function and no active bleeding, on regular prophylactic recombinant factor VIII concentrate substitution (2500 IU every 48 h via porta-cath due to poor peripheral venous access).

Initial ultrasound of multiple joints revealed moderate synovial hypertrophy in the dorsal aspect of the left radial-carpal and wrist joints, as well as the right ankle joint. Uneven articular cartilage outline in the left elbow joint. The HEAD-US score was 4. In full blood count iron deficiency anemia was detected and oral supplementation with iron was initiated, control factor VIII inhibitor was negative.

### 2.4. Parameters Measured

#### 2.4.1. Body Composition (InBody 270)

The InBody 270 analyzer (InBody Co., Ltd., Seoul, Republic of Korea) using bioelectrical impedance analysis (BIA) technology to accurately assess various aspects of a body composition was employed in this study. Using an advanced methodology based on multiple current frequencies, the InBody allows precise differentiation between different body components such as fat, muscle, bone and water [20,21]. During the measurements, patients were asked to stand on the scale in designated areas, and prohibited from moving during the analysis.

#### 2.4.2. Hemophilia Joint Health Score

The HJHS assesses the health of the joints that are most commonly affected by bleeding in patients dealing with hemophilia: ankle joints, knee joints, elbow joints. HJHS is tailored to evaluate children (4–18 years old) with hemophilia and mild joint impairment and adult. It can also be used in orthopedic procedures or as a measuring tool during physiotherapy visits. Three pediatric studies have demonstrated good inter-rater reliability, with Intraclass Correlation Coefficients (ICC) exceeding 0.70, indicating acceptable agreement between different examiners. The HJHS was designed to be sensitive to subtle changes in joint health, especially in countries with preventive treatment and when there is subtle evidence of joint damage. In studies, compared to World Federation of Hemophilia WFH, HJHS was 97% more effective in differentiating severe hemophilia from mild and moderate hemophilia, and 74% more effective than WFH in patients receiving prophylaxis versus on-demand treatment [22].

The current version, HJHS 2.1, is based on an assessment of swelling (0–3); swelling duration (0–1); muscle atrophy (0–2); snapping during movement (0–2); loss of flexion (0–3); loss of extension (0–3); joint pain (0–2) and strength (0–4) of knees, ankles and elbows; and overall gait score (0–4). A higher score indicates lower joint health.

#### 2.4.3. Joint Ultrasound (HEAD-US Scoring)

The ultrasound examination was performed after the physical evaluation, during the same visit, according to the guidelines of the HEAD-US protocol [23]. This method was chosen because of the recommendations of specialists who recommend its use in daily clinical practice [24]. The HEAD-US protocol was found to be an effective, reliable and accurate technique for diagnosing and monitoring HA. It has a high sensitivity in detecting changes involving the synovial membrane and osteochondral structures, with values ranging from 0.92 to 0.97 compared to results obtained with magnetic resonance imaging (MRI), depending on the series of tests analyzed [12,25]. The elbow, knee and ankle joints were evaluated during the study, with points assigned based on the degree of synovial hypertrophy (0–2), cartilage damage (0–4) and subchondral bone changes (0–2). The maximum score for a single joint was 8, where a higher score indicated greater severity of pathology. Additionally, the same radiologist performed a supplementary qualitative scan of other clinically relevant joints (shoulder, wrist) to identify possible abnormalities, but these observations were not included in the HEAD-US scoring. The same ultrasound apparatus was used for all studies, and the same radiologist. Dedicated radiologist experienced in musculoskeletal assessment performed all the examinations with the same ultrasound apparatus.

#### 2.4.4. Quality of Life (EQ-5D-5L)

Quality of life was assessed using the validated EQ-5D-5L instrument. It consists of five dimensions: mobility, self-care, performance of usual activities, pain/discomfort and anxiety/depression, with five levels of severity of problems. In the context of patients with severe hemophilia, the use of the EQ-5D-5L scale allows a detailed analysis of the impact of physiotherapy, including resistance training and manual therapy, on their quality of life [26]. For each participant, both the five-digit health profile (descriptive system) and the EQ-5D index value were documented to allow qualitative interpretation relative to normative population values. ICC from previous studies ranged from 0.65 to 0.91 indicating good measurement reliability [27]. MCID values for EQ-5D-5L varied, ranging from 0.03 to 0.52. In addition, participants rated their overall health on a Visual Analogue Scale (VAS) from 0 (worst imaginable health) to 100 (best imaginable health). The patient determines the intensity of pain by marking a point on the section that corresponds to how they feel, significant changes are considered when ranged from 6.5 to 8.2 points [28]. For practicality, only the EQ-VAS component was repeated post-intervention to capture short-term perceived health changes. The full five-dimension EQ-5D-5L profile and corresponding index value were obtained only at baseline to avoid participant fatigue.

#### 2.4.5. Static Balance (One-Leg Standing Test, OLS)

The One Leg Standing (OLS) test was used to evaluate static balance in two conditions: with eyes open (EO) and with eyes closed (EC). Participants stood barefoot on one leg with the opposite knee flexed, avoiding contact with the supporting leg. Their arms were crossed over the chest to eliminate arm compensation. In the EO condition, subjects were instructed to fix their gaze on a point at eye level, while in the EC condition, visual input was removed to increase reliance on proprioceptive and vestibular feedback. Each condition was tested three times, and the average duration (in seconds) of the three trials was used for analysis. A rest interval of up to 2 min was provided between trials to minimize fatigue [29,30]. The OLS is considered a highly reliable tool for assessing balance, especially when standardized procedures are used. For EO trials ICC ranges from 0.85 to 0.95, indicating very high reliability. For EC ICC ranges from 0.78 to 0.90, indicating good to very good reliability. In the general population, a change of 3–5 s is considered clinically significant.

#### 2.4.6. Ankle Dorsiflexion (Weight-Bearing Lunge Test, WBLT)

The WBLT was used to assess the range of ankle dorsiflexion (ankle DF) under load. The participant stood facing the wall in a pronated position, keeping the heel of the test foot flat on the ground [31]. The participant’s task was to touch the wall with the knee, without taking the heel off the ground. The distance between the big toe of the foot and the wall was measured with a ruler or yardstick to assess the range of motion. The test was performed three times, and the average value of the three trials was used for analysis. The range of dorsiflexion considered correct is at least 12.5 cm of distance from the wall. The test shows high reliability, between evaluators (ICC = 0.80–0.99), within the same evaluator (ICC = 0.65–0.99). In addition, a MDC of approximately 1.9 cm has been established for the WBLT [32].

#### 2.4.7. Joint Range of Motion (SFTR Method)

The SFTR (Sagittal-Frontal-Transverse-Rotation) system accurately quantifies joint angular ranges of motion and is also referred to as the ISOM (International Standard Orthopedic Measurements) method. Measurements are recorded using a three-digit format based on the following conventions. First digit: Extension, external rotation, retraction, inversion, lateral bending, and left rotation. Middle digit: Neutral starting position, typically zero under physiological conditions. Last digit: Flexion, internal rotation, adduction, eversion, lateral obliques, and right rotation [33].

Joint mobility is assessed from a neutral zero position, allowing for the evaluation of both active and passive range of motion. Studies have demonstrated that goniometric ROM (Range of Motion) assessments, including the SFTR method, exhibit good to excellent reliability. In our study, the SFTR method was employed to assess joint range of motion in patients with severe hemophilia, enabling the monitoring of therapeutic progress and rehabilitation effectiveness. Due to the absence of clearly defined MCID values for SFTR measurements, result interpretation required an individualized approach, considering each patient’s clinical condition [34].

#### 2.4.8. Isometric Muscle Strength (Hand-Held Dynamometry)

Muscle strength was assessed using a Lafayette Manual Muscle Testing (MMT) dynamometer (Lafayette Instrument Company, Lafayette, IN, USA), which measures peak force output in kilograms. Measurements were conducted in a seated position, with the elbow flexed at 90 degrees for assessing biceps and triceps strength, and the knee flexed at 90 degrees for evaluating quadriceps and hamstring strength. The dynamometer was positioned just proximal to the wrist for elbow assessments and just proximal to the ankle for knee measurements. Each test was repeated three times, with the highest value recorded for analysis, ensuring accuracy and reliability. In this study, muscle strength assessment with the Lafayette dynamometer was crucial for monitoring rehabilitation progress in patients with severe hemophilia, where joint damage and recurrent hemarthrosis contribute to muscle weakness. The results were analyzed alongside joint range-of-motion measurements to provide a comprehensive evaluation of musculoskeletal function and therapy effectiveness.

To minimize variability, a standardized protocol was followed, including consistent patient positioning, a 30 s rest between trials, and verbal encouragement to ensure maximal effort. All tests were conducted by the same examiner to enhance reproducibility. The Lafayette dynamometer has demonstrated high intra- and inter-rater reliability. Its high reliability in measuring hip muscle strength and pain thresholds has been confirmed in previous studies [35,36]. In a reliability study by Mentiplay, the use of a hand dynamometer to assess lower limb muscle strength yielded ICC values ≥ 0.70 across different muscle groups, suggesting good to excellent measurement consistency [37].

#### 2.4.9. Blood Sample Collection and Immunological Analyses

After qualification of patients with hemophilia, blood samples were collected to assess the concentration of neuroinflammatory factors including (IL-18), (β-NGF), (SsICAM-1), (sRAGE), (CCL2). These biomarkers were selected to reflect chronic low-grade inflammation, endothelial activation, and neurotrophic signaling relevant to hemophilic arthropathy. Blood was collected twice from each patient (pre-intervention and post-intervention). Reference intervals for IL-18, β-NGF, ssICAM-1, sRAGE and CCL2 were taken from previously collected laboratory data of healthy children examined at the University Clinical Center of Gdańsk (UCK) for assay validation purposes. These children were not enrolled as a control group in the present study.

Serum was separated by centrifugation at 4500× *g* for 10 min and stored at −80 °C at the Department of Immunobiology and Environmental Microbiology, Medical University of Gdańsk, until analysis. Biomarker levels were measured using commercial ELISA kits (Bioassay Technology Laboratory, Jiaxing Korain Biotech Co., Jiaxing, China; kit numbers: E4729Hu, E0147Hu, E0764Ra, E2102Hu, E0017Ca), following the manufacturer’s protocol. Optical density (OD) was determined using a ChroMate 4300 Microplate Reader (Awareness Technology, Palm City, FL, USA) at 450 nm. All assays were performed in technical duplicates, ensuring high reliability (inter- and intra-assay CV < 5%).

### 2.5. Physiotherapy Intervention

#### 2.5.1. Progressive Resistance Training Program

The program was tailored to each participant’s capabilities, aiming to support muscle strength and joint stabilization. Resistance was gradually increased, and exercises included both isometric and dynamic movements using elastic bands or light training equipment. The 8-week intervention included twice-weekly, 60 min sessions, emphasizing proper technique to minimize injury risk. In each session, exercises were performed in a fixed sequence, moving from one exercise to the next. Each patient underwent educational training to ensure the correct technique for each exercise (Table 1). The warm-up (10 min) consisted of marching in place, arm circles, knee raises, and hip rotations.

Each exercise was performed for 3 sets with fixed rest (60 s) and controlled tempo (1 s concentric/3 s eccentric). Progression followed a pre-planned rep scheme: weeks 1–2: 3 × 20 reps; weeks 3–4: 3 × 15; weeks 5–6: 3 × 12; weeks 7–8: 3 × 8. Load (elastic band tension or dumbbell weight) was adjusted so that the participant could complete the target reps with ~1 repetition in reserve (RIR ≈ 1). A preliminary trial set was used to verify load selection; if the target repetitions could not be achieved with proper form, the load was reduced or the exercise regressed (e.g., assisted squats using a ladder). To achieve the appropriate exercise intensity, a trial set was conducted using either a black resistance band or a 1 kg dumbbell. If necessary, the load was adjusted to meet the required intensity. When participants were unable to complete the target repetitions, regression techniques were applied, such as using a ladder for assisted squats. Resistance bands of varying tensions (blue, light green, yellow) were used either individually or in combination. Dumbbells ranged from 1 to 15 kg, with fine adjustments in increments of 0.5 kg. Each participant had an individually tailored intensity and exercise pace to ensure proper execution of movements within their maximum ROM. Lifting speed was variable, with 1 s for the concentric phase and 3 s for the eccentric phase of each task RM (Repetition Maximum) values were continuously monitored to ensure maximum repetitions were achieved without compromising form or safety.

#### 2.5.2. Manual Therapy Protocol

Manual therapy was performed by a licensed physiotherapist using standardized joint traction techniques, consistent with previously described approaches in hemophilia rehabilitation [38]. Each session targeted the joint with the highest HJHS score (most affected joint), typically the knee or ankle. Belt-assisted traction was applied in a pain-free neutral position, with Grade I–II distraction force (joint surface separation without tissue strain), aiming to reduce compression, relieve pain, and improve capsular mobility.

For each target joint, three 60 s tractions were performed, separated by 60 s rest intervals. The physiotherapist continuously monitored end-feel quality, joint play, and patient comfort to ensure safe and reproducible dosing. Sessions were conducted at the end of each physiotherapy visit to promote post-exercise relaxation and optimize recovery. Traction was applied in submaximal ranges of motion, avoiding joint stress or hemarthrosis risk. The patient was positioned supine, with the distal tibia stabilized and the talus gently distracted using a belt and manual support.

#### 2.5.3. Safety Monitoring

Participants’ safety was closely monitored throughout the study using daily hemarthrosis assessments conducted by parents or guardians at home, with verification by the therapeutic team during follow-up visits. Prophylactic administration of clotting factors (factor VIII or IX) in patients 1, 3 and 4 was provided 2–3 h before each physiotherapy session, patient receiving prophylaxis with emicizumab had no factor infusions before sessions according to the hemophilia prophylaxis guidelines. This monitoring framework enabled a detailed evaluation of the individualized physiotherapy program’s impact on key clinical and functional outcomes in patients with severe hemophilia while ensuring optimal safety. The methodology adheres to best-practice standards for managing hemophilia [39]. Participants and their parents were instructed to avoid contact or high-impact sports and activities carrying an elevated risk of trauma during the intervention period. However, no systematic monitoring of extracurricular physical activity (e.g., school physical education or recreational exercise) was performed, reflecting the exploratory nature of this case series.

### 2.6. Data Analysis

Due to the small sample size (*n* = 4) and the descriptive nature of this case series, no formal statistical testing was conducted. All variables were summarized descriptively at the individual level (pre- and post-intervention). Changes within each case were expressed as raw values and percentage differences to illustrate trends rather than to infer statistical significance.

## 3. Results

### 3.1. Participant Characteristics and Laboratory Findings

Baseline anthropometric and laboratory data for the four participants are presented in Table 2. All patients were in good general condition, with stable factor prophylaxis and no active bleeding at baseline. Routine biochemical and hematological parameters were largely within reference limits. Mild leukopenia was noted in Cases 1–3 (2.2–3.5 × 10^9^/L), though these findings were clinically asymptomatic and not associated with infection, medication effects, or hematologic disorders. Vitamin D concentrations (25-OH) ranged between 25.5 and 29.4 ng/mL in all participants at both assessments, remaining slightly below the optimal range for adolescents (≥30 ng/mL). No systematic vitamin D supplementation was prescribed as part of the study protocol, and participants continued their standard nutritional routines. Liver and renal function tests remained normal across all participants. No relevant abnormalities were detected that would contraindicate exercise participation.

### 3.2. Hemophilia Joint HealthScore—HJHS

Pre- to post-intervention results are summarized in Table 3. All participants demonstrated improvements in overall joint health, with total HJHS scores decreasing by 35–62%. The largest functional gains were observed in the knee and ankle joints, reflecting reduced stiffness and pain after the 8-week physiotherapy program. Minor fluctuations in subscores (e.g., a small knee increase in Case 1) were clinically transient and not accompanied by structural deterioration on ultrasound. These consistent HJHS reductions indicate better joint function and support the feasibility of individualized exercise and manual therapy in adolescents with severe hemophilia.

### 3.3. Quality of Life EQ-5D-5L QOL

Only EQ-VAS scores were reassessed after the intervention; the EQ-5D-5L index was collected at baseline. As shown in Table 4, quality-of-life scores (EQ-VAS) improved in three of four participants, with relative increases ranging from 2% to 15%. The most consistent improvements were noted in mobility and pain/discomfort dimensions, while self-care and anxiety/depression remained stable. One participant maintained a high baseline score without meaningful change. Overall, these findings indicate small-to-moderate perceived gains in daily functioning and comfort, consistent with the improvements observed in joint function and strength measures.

### 3.4. Upper and Lower Limb Isometric Strength

The results of the isometric strength assessments with Lafayette Dynamometer showed improvements across multiple joints, with variability depending on the joint and participant. Case 1 showed a 23.4% increase in left elbow strength, rising from 17.95 kg to 22.15 kg, while right elbow strength increased by 3.8% (17.25 kg to 17.90 kg). Case 2 demonstrated greater gains, with left elbow flexion strength increasing by 38.0% (24.75 kg to 34.15 kg) and right elbow strength improving by 13.2% (25.35 kg to 28.70 kg). Elbow extension strength also improved significantly, with Case 1 exhibiting a 49.2% gain in left elbow extension (12.10 kg to 18.05 kg) and a 25.5% increase in right extension (13.55 kg to 17.00 kg). For knee extension, Case 1 displayed a notable improvement in right knee strength, increasing by 70.2% (20.30 kg to 34.55 kg). However, Case 2 experienced a slight decrease in right knee extension by 3.1% (28.95 kg to 28.05 kg). Ankle dorsiflexion and plantarflexion strength exhibited variable results across cases.

#### Target Joint Strength

In Case 1, right ankle dorsiflexion strength improved from 11.35 kg to 15.40 kg, reflecting a 35.7% increase, while plantarflexion showed a substantial gain from 8.50 kg to 17.70 kg, marking a 108.2% improvement. In Case 2, left ankle dorsiflexion exhibited a slight decrease from 14.15 kg to 12.90 kg (−8.8%), but plantarflexion demonstrated a large increase from 13.75 kg to 36.70 kg (+166.9%). For Case 3, right ankle dorsiflexion improved significantly from 9.15 kg to 16.00 kg, representing a 75.96% increase, while plantarflexion strength increased from 14.10 kg to 33.35 kg, resulting in a 136.5% gain. In Case 4, right ankle dorsiflexion increased from 10.15 kg to 16.75 kg (+65%), and plantarflexion strength rose from 12.90 kg to 30.85 kg (+139.1%).

### 3.5. Range of Motion

Table 5 shows the ranges of motion of the two joints that had the worst results before and after therapeutic intervention. The study concerns the elbow and ankle joints. The results show an increase in the range of motion after the therapy, the joint undergoing target joint manual therapy was marked in red.

### 3.6. Ankle Dorsiflexion (WBLT)

The WBLT results demonstrated varied small responses across participants in both right and left ankles. Case 1 showed a 6.67% increase in right ankle dorsiflexion from 7.5 cm to 8.0 cm, while the left ankle remained stable at 8.0 cm with no measurable change. Case 2 experienced a decrease of 7.14% in right ankle dorsiflexion from 7.0 cm to 6.5 cm, with no changes observed in the left ankle. In Case 3, right ankle dorsiflexion also declined by 7.14%, decreasing from 7.0 cm to 6.5 cm, while the left ankle improved by 66.67%, increasing from 1.5 cm to 2.5 cm. Case 4 maintained stability in right ankle dorsiflexion with no change from 8.5 cm, while the left ankle showed an 18.75% increase, improving from 8.0 cm to 9.5 cm.

### 3.7. Static Balance

For the right and left lower limb stance with eyes open, there was no change across participants, maintaining stability at 60 s (maximum value). Single leg stance with eyes closed demonstrated improvements in both lower-limbs in all cases. Case 1 showed a 62.2% increase in duration, from 37 s to 60 s. Case 3 exhibited a 36.4% improvement, increasing from 44 s to 60 s, while Case 4 demonstrated the most pronounced gain of 150%, improving from 16 s to 40 s.

### 3.8. Joint Imaging: HEAD-US and Additional Ultrasound Findings

According to the HEAD-US protocol, only the elbow, knee, and ankle joints were scored. Additional qualitative ultrasound assessments of other joints (e.g., wrist, shoulder) were performed outside the scoring system to document any co-existing abnormalities but were not included in the HEAD-US score. In Case 1, the HEAD-US assessment showed localized cartilage irregularities in the left knee and both ankles (score = 1 per joint). During supplementary scanning, moderate synovial hypertrophy was also observed in the left wrist and mild irregularities in the right shoulder (descriptive findings only). Case 4 presented mild synovial hypertrophy in the right ankle and cartilage irregularities in the left elbow (HEAD-US = 1 each). Qualitative examination additionally revealed moderate synovial hypertrophy in the left wrist (not scored). No joint deterioration was detected in any participant after the physiotherapy intervention.

### 3.9. Analysis of Chosen Biomarkers

The analyzed biomarkers included interleukin-18 (IL-18), β-nerve growth factor (β-NGF), intercellular adhesion molecule-1 (sICAM-1), soluble receptor for advanced glycation end-products (sRAGE), and C-C motif chemokine ligand-2 (CCL2). Baseline and post-intervention values are presented in Table 6, together with reference ranges obtained from healthy pediatric samples used for assay validation. Across participants, IL-18 and sICAM-1 consistently decreased after the 8-week intervention, suggesting attenuation of pro-inflammatory and endothelial-activation processes. β-NGF and sRAGE generally increased, indicating possible neurotrophic and compensatory responses, while CCL2 changes were heterogeneous (decrease in one participant, mild increases in others). In two participants (Cases 3 and 4), IL-18 and sICAM-1 concentrations declined from clearly elevated to within or near normal limits, which may reflect a biologically relevant improvement in inflammatory status.

## 4. Discussion

The main aim of this case series was to describe the short-term outcomes of an eight-week rehabilitation program combining resistance training and manual therapy in adolescents with severe hemophilia. The program was designed to support joint function, enhance muscle strength, and document changes in inflammatory biomarkers associated with hemophilic arthropathy.

The observations suggest that individualized rehabilitation may contribute to functional improvements across several domains. Although overall HJHS scores improved in all participants, one case (Case 1) showed a slight increase in knee subscores despite total score reduction. This minor fluctuation most likely reflected transient stiffness or local overload adaptation during the early training phase rather than true arthropathy progression, as no new lesions were observed on ultrasound. Overall, the observed HJHS reductions represent individual short-term responses within this series rather than conclusive evidence of therapeutic efficacy. Similar observations were reported by Fischer et al. [24], who showed that HJHS can detect therapy-related changes and help differentiate prophylactic strategies in patients with varying severity of hemophilia. In addition, validation data indicate that HJHS is useful for monitoring joint status in clinical practice, including adults [13,22]. Nevertheless, the time burden in outpatient settings and dependence on examiner experience have been highlighted as limitations [12,24,25,40].

With respect to range of motion, our study using the SFTR method showed measurable improvements, confirming its utility in monitoring rehabilitation progress. More recent standardized approaches (e.g., KRSP-type protocols) appear to provide higher inter-examiner consistency and lower error than traditional goniometry, which future studies should consider [41].

Analysis of muscle strength with the Lafayette dynamometer indicated clear gains in most patients. High reliability of this tool in musculoskeletal disorders has been demonstrated [35], supporting our methodological choice. However, in Case 2 a slight decrease in right knee extensor strength was observed (–3.1%) despite improvements in other joints. This may reflect preferential loading due to contralateral arthropathy and asymmetric training response. A similar pattern appeared for ankle dorsiflexion in Case 2 (modest decline with marked plantarflexion improvement), underscoring the need to individualize loads when joint involvement is asymmetric. Independent work has also confirmed the instrument’s reliability in related pressure-threshold applications [36].

Consistent improvements were also noted in balance and stability tests (OLS) and in ankle dorsiflexion assessed with the weight-bearing lunge test. Importantly, HEAD-US ultrasound revealed no progression of arthropathy, suggesting that the intervention was safe and may help stabilize joint structures in the short term.

In addition to functional outcomes, this study explored biochemical markers of inflammation and neurotrophic activity. We observed a reduction in IL-18 and sICAM-1 together with an increase in β-NGF and, in most cases, sRAGE. These results align with findings by Wehmeier et al. [16], who reported elevated IL-18 in hemophilia and emphasized its role in neuroinflammation and disease progression; experimental IL-18 blockade reduced neuropathic pain and increased opioid efficacy. Furthermore, sICAM-1 has been linked to endothelial dysfunction in hemophilia [42], which makes the observed post-therapy decrease clinically meaningful. Animal studies also demonstrated increased CCL2 in factor VIII-deficient models, confirming its role in joint inflammation [14]—although in our cohort CCL2 decreased only in one case, which warrants further investigation. The observed increase in β-NGF may suggest compensatory neuroprotective effects and is consistent with reports in sickle-cell disease, where β-NGF levels negatively correlated with pain severity [18]. Taken together, these biomarker trends are exploratory and hypothesis-generating, suggesting potential anti-inflammatory and neuroprotective responses that merit further controlled investigation [14,16].

The rationale for combining resistance and manual therapy in our program was based on existing evidence that structured, supervised exercise is both feasible and beneficial in hemophilia. Programmed Sports Therapy (PST), introduced by Hilberg et al. [6], demonstrated that multi-component exercise integrating strength, coordination, and endurance training can enhance physical function and slow arthropathy progression. Similarly, Wilczyński et al. [7] reported that progressive resistance training was safe even in patients with inhibitors when conducted under adequate prophylaxis. Furthermore, systematic reviews in healthy and clinical populations suggest that moderate training volume (12–20 weekly sets) is sufficient for muscle hypertrophy and neuromuscular adaptation without excessive load [8]. These findings collectively support our choice of moderate-intensity, multi-component exercise dosing, balancing therapeutic efficacy with bleeding risk in adolescents with severe hemophilia.

When compared with previous reports, our results are consistent with those of Hilberg [6], who demonstrated that tailored sports therapy can slow joint degeneration, and with Cuesta-Barriuso [11] and Scaturro et al. [41], who confirmed the benefits of manual therapy and home exercises on mobility and pain reduction. Similarly, Schäfer et al. [43] emphasized the importance of physical activity in maintaining musculoskeletal health in hemophilia. The present case series contributes to this evidence base by illustrating consistent individual-level trends across functional and biomarker domains, providing preliminary descriptive data that may guide future research design.

### 4.1. Limitations

Despite the promising results, several limitations should be acknowledged. The sample size was small (*n* = 4), and no contemporaneous control group was included; instead, reference biomarker ranges were derived from healthy pediatric samples previously collected at our institution, which limits generalizability. The participants’ young age and the heterogeneity in prophylactic regimens (factor VIII, factor IX, or emicizumab) may also have influenced the outcomes. Although no major intraarticular hemorrhages were observed, subclinical bleeding events cannot be excluded. Furthermore, the 8-week intervention period was relatively short, precluding conclusions about long-term effects. The duration of the observed functional and biochemical improvements remains unknown, as no post-intervention follow-up was performed. Extracurricular activity levels were not standardized or continuously monitored, which may have influenced individual functional outcomes. Given the descriptive case-series design, all findings should be interpreted as observational and hypothesis-generating rather than confirmatory. Future research should include a larger cohort, matched controls, and follow-up assessments at six and twelve months to evaluate the durability of benefits and the potential need for cyclical interventions.

### 4.2. Practical Implications

From a clinical standpoint, this case series provides descriptive but meaningful evidence that supervised resistance training combined with gentle manual therapy can be safely integrated into rehabilitation programs for youth with severe hemophilia. Tailoring exercise intensity and modality to the patient’s bleeding history and joint status is crucial, as shown by the differences between patients in this study. The literature, including the systematic review by Baz-Valle et al. [8], also emphasizes the importance of individualized resistance training to optimize outcomes. Incorporating physiotherapy early in multidisciplinary hemophilia management could help prevent secondary joint deterioration and support long-term musculoskeletal health. Future studies with larger cohorts and extended follow-up are warranted to confirm these benefits and define optimal dosing and frequency of physiotherapy interventions.

## 5. Conclusions

This case series describes short-term functional and biochemical outcomes following an eight-week physiotherapy program combining resistance training and manual therapy in adolescents with severe hemophilia. Observed trends suggested potential improvements in joint function, muscle strength, and pain perception, accompanied by changes in inflammatory biomarkers, including IL-18 and β-NGF, that may reflect anti-inflammatory or neuromodulatory responses. The combined use of functional testing and imaging (HEAD-US) was feasible for documenting joint status within individualized rehabilitation. Overall, these descriptive findings highlight the potential role of structured physiotherapy as a supportive component of hemophilia care and underscore the need for larger, controlled, and longitudinal studies to confirm and expand these preliminary observations.

## Figures and Tables

**Table 1:** Progressive Resistance Training.

Exercise Name	Short Description	Sets	Repetitions	Tempo	Rest Time	Intensity/Progression
Good morning with resistance band	This exercise strengthens the posterior chain, including the hamstrings, glutes, and lower back. It is a safe alternative to the barbell version.	3	20/15/12/8	3010	60 s	individually tailored intensity and progression (Supplementary Materials)
One-arm row with resistance band	This exercise strengthens the back muscles, particularly the latissimus dorsi and biceps strength and improving posture.					
Dumbbell bench press	An exercise that develops the chest, triceps, and shoulder muscles. It helps build upper body strength and endurance.					
Squats	A fundamental exercise that strengthens the lower body, including the quadriceps, glutes, and hamstrings.					
Single-leg stance with lateral raises	An exercise that enhances balance, stabilization, and strengthens the shoulders and legs					
Forward lunges	This exercise strengthens the leg and glute muscles while improving stability and mobility					
Overhead press with resistance band	An exercise that strengthens the shoulders and arms, enhancing upper body endurance					

Legend. Rest time refers to rest between series. Short description—Provides a brief explanation of the exercise, including its benefits and targeted muscle groups. Sets—Refers to the number of times a full sequence of repetitions is performed. Repetitions—Refers to the number of times an individual movement is repeated within a set. Tempo—Refers to the speed at which the movement is performed, typically including eccentric (lowering) and concentric (lifting) phases. Rest Time—Refers to the duration of rest between sets or exercises to allow for recovery. Intensity/Progression—Refers to the method of increasing the difficulty of the exercise, such as adding resistance, increasing repetitions, or adjusting tempo. Execution is available in Supplementary Materials.

**Table 2:** Baseline anthropometric and laboratory characteristics of the four participants.

Parameter	Case 1		Case 2		Case 3		Case 4	
Age (years)	17		16		11		15	
Diagnosis	Hemophilia A (severe)		Hemophilia A (severe, with inhibitor)		Hemophilia B (severe)		Hemophilia A (severe)	
Prophylaxis	Factor VIII 2000 IU, 3×/week		Emicizumab (Hemlibra)		Factor IX (Benefix) 2750 IU, 3×/week		Factor VIII (Novoeight) 2500 IU, every 48 h	
Target joint	right ankle		left ankle		right ankle		right ankle	
Weight (kg)	57.4		79.1		50.9		76.6	
Height (m)	1.67		1.75		1.49		1.69	
BMI (kg/m^2^)	20.6		25.8		22.9		26.8	
Lean mass (kg)	29.3		30.2		18		31.4	
Body fat (%)	9.5		32.4		33.7		27.3	
Parameters pre and post intervention	Case 1 (Pre)	Case 1 (Post)	Case 2 (Pre)	Case 2 (Post)	Case 3 (Pre)	Case 3 (Post)	Case 4 (Pre)	Case 4 (Post)
Hemoglobin [g/dL]	14.6	14.9	15.5	16.1	11.7	11.9	14.3	13.8
Hematocrit [%]	42.6	44.5	45.4	47.6	36.1	35.1	44.2	42.3
MCV [fL]	83.5	84.8	84.2	85.3	81.9	80.7	78.2	80.4
MCH [pg]	28.6	28.4	28.8	28.9	26.5	26.9	25.3	26.2
MCHC [g/dL]	34.3	33.5	34.1	33.8	32.4	33.3	32.4	32.6
Leukocytes [×10^9^/L]	2.21	1.42	2.80	3.21	3.47	4.35	6.16	5.26
Lymphocytes [%]	42.6	40.5	37.9	34.6	31	30.8	20.8	16.7
Neutrophils [%]	45	51.1	49.9	52.2	55.9	55.9	70.9	73.7
Monocytes [%]	9.4	8.9	8.2	8.6	8.9	8.6	6	7.3
Reticulocytes [%]	1	1	1.5	1.8	1.1	1.5	1.2	1.1
CRP [mg/L]	0.4	0.5	0.7	0.8	5	4	0.41	0.41
Creatinine [mg/dL]	0.87	0.92	0.89	0.84	0.47	0.52	0.76	0.91
Urea [mg/dL]	14	15	10	7	15	13	9	8
ALT [U/L]	15	13	36	34	20	24	13	15
AST [U/L]	22	24	34	35	28	31	17	21
Bilirubin Total [mg/dL]	1	0.83	1.1	0.94	0.60	0.46	1	0.49
Albumin [g/L]	49	55	44	48	45	46	46	49
TSH [uU/mL]	0.6	1.3	0.7	1.2	0.925	1.140	1	1.151
Parathormone [pg/mL]	10.3	13.5	19.7	20.7	12.7	14.2	21.6	20.4
Vitamin D (25-OH) [ng/mL]	25.8	28.7	25.5	25.8	26.8	29.4	25.8	26.8
Vitamin B12 [pg/mL]	437	486	477	522	522	553	312	320
Folic Acid [ng/mL]	4.8	5.1	4.9	6.3	6.3	6.7	12.2	11.7
Ferritin [ng/mL]	51.57	51.74	54.25	60.51	60.57	60.14	18.9	18.49
Calcium Total [mg/dL]	9.9	9	9.8	9.9	9.2	9.5	9.7	9.8
Uric Acid [mg/dL]	6.7	6.9	5.7	6.2	3.2	3.5	6	6.2

Baseline (abbreviations): ABR—Annual Bleeding Rate; JABR—Joint Annual Bleeding Rate; HEAD-US—Hemophilia Early Arthropathy Detection with Ultrasound; HJHS—Hemophilia Joint Health Score (version 2.1). Reference ranges vary by laboratory and age; therefore, deviations (↑/↓) are not indicated in the table to avoid misinterpretation. All values were interpreted relative to pediatric reference intervals from the University Clinical Center laboratory. Three participants showed mild leukopenia (2.2–3.4 × 10^9^/L) at baseline, which was clinically asymptomatic and did not contraindicate participation.

**Table 3:** HJHS 2.1 scores pre- and post-intervention with percentage change.

Participants		HJHS (Total Score)	% Change	Elbow		Knee		Ankle	
				Left	Right	Left	Right	Right	Left
Case 1	Pre	14.00	−35.7	0	0	1	2	6	4
	Post	9.00		0	0	2	3	2	2
Case 2	Pre	13.00	−61.5	0	1	4	1	1	5
	Post	5.00		0	0	2	0	1	2
Case 3	Pre	16.00	−56.2	3	3	1	1	3	4
	Post	7.00		0	0	0	3	1	2
Case 4	Pre	15.00	−40.0	2	2	3	2	2	5
	Post	9.00		1	0	0	1	2	3

Abbreviations: HJHS—Hemophilia Joint Health Score (version 2.1). Scoring: 0 = no impairment; 1 = minimal/mild symptoms (e.g., slight stiffness or pain); 2 = mild–moderate symptoms; 3 = moderate joint dysfunction (e.g., limited range of motion, swelling); 4 = severe joint damage with marked limitation; 5 = extreme joint damage; 6 = deformity.

**Table 4:** Quality of Life EQ-5D-5L scores before and after intervention among participants.

Participants		EQ VAS	% Change	EQ-5D-5L	Mobility	Self-Care	Usual Activities	Pain/Discomfort	Anxiety/Depression
Case 1	Pre	90	2.22	11121	1	1	1	2	1
	Post	92		11121	1	1	1	2	1
Case 2	Pre	80	12.5	21221	2	1	2	2	1
	Post	90		11111	1	1	1	1	1
Case 3	Pre	89	2.25	11311	1	1	3	1	1
	Post	91		11111	1	1	1	1	1
Case 4	Pre	80	15.0	11311	1	1	3	1	1
	Post	92		11212	1	1	2	1	2

Abbreviations: EQ-5D-5L—EuroQol 5-Dimension 5-Level questionnaire; EQ VAS—EuroQol Visual Analogue Scale. Scoring: 1 = no problems; 2 = slight problems; 3 = moderate problems.

**Table 5:** Range of motion test results of the 2 joints performing worst in mobility limitation and the worst HJHS score.

Patient	Joint with Most Limited ROM	ISOM Norms	Pre	Post
Case 1	left ankle	20-0-45	23-0-25	23-0-30
Target Joint	right ankle	20-0-45	20-0-25	26-0-37
Case 2	right ankle	20-0-45	17-0-44	19-0-45
Target Joint	left ankle	20-0-45	10-0-45	15-0-45
Case 3	left elbow	0-0-150	5-0-140	0-0-140
Target Joint	right ankle	20-0-45	16-0-35	16-0-40
Case 4	left elbow	0-0-150	5-0-130	0-0-145
Target Joint	right ankle	20-0-45	20-0-25	20-0-40

Abbreviations: ROM—Range of Motion; HJHS—Hemophilia Joint Health Score; ISOM Norms—Standard reference values for isolated joint movement (in degrees). Notation: Flexion–Extension expressed as X–0–Y, where X = extension deficit, 0 = neutral position, and Y = flexion range.

**Table 6:** Comparison of the chosen biomarker levels pre- and post- intervention in comparison to healthy children without hemophilia.

Biomarker	Normal Range	Reference Ranges Are Derived from Previously Collected Healthy Pediatric Samples from UCK (*n* = 8)	Case	Pre-Intervention	Post-Intervention	Change (%)	Interpretation
IL-18	100–300 pg/mL	174.52	1	125.14	109.16	↓ 12.77%	Within normal; modest decrease
			2	199.56	105.61	↓ 47.08%	Within normal; significant decrease
			3	631.45	245.34	↓ 61.15%	Elevated → normal; strong improvement
			4	410.18	294.91	↓ 28.11%	Elevated → borderline high
ß-NGF	10–50 pg/mL	45.50	1	52.14	56.41	↑ 8.19%	Slightly high → further increase
			2	36.25	44.12	↑ 21.71%	Within normal; healthy increase
			3	15.12	29.65	↑ 96.10%	Within normal; strong beneficial increase
			4	24.78	32.45	↑ 30.98%	Within normal; notable increase
sICAM-1	150–400 ng/mL	144.65	1	254.52	175.12	↓ 31.20%	Within normal; healthy decrease
			2	163.45	158.21	↓ 3.21%	Within normal; stable
			3	741.95	302.32	↓ 59.36%	Elevated → normal; strong improvement
			4	532.22	421.56	↓ 20.79%	Elevated → borderline high
sRAGE	800–1400 pg/mL	1362.25	1	1321	1458	↑ 10.37%	Normal → slightly high; potentially beneficial
			2	897	968	↑ 7.92%	Normal; healthy increase
			3	687	641	↓ 6.70%	Below normal; slight drop
			4	1235	1413	↑ 14.41%	Normal → slightly high
CCL2	100–150 pg/mL	105.45	1	174.15	179.12	↑ 2.85%	Elevated → further elevation
			2	149.16	161.23	↑ 8.09%	High end of normal → elevated
			3	304.55	164.28	↓ 46.11%	Very elevated → moderate high
			4	182.12	198.43	↑ 8.95%	Elevated → further elevation

Abbreviations: IL-18—Interleukin-18; β-NGF—Beta-Nerve Growth Factor; sICAM-1—Soluble Intercellular Adhesion Molecule-1; sRAGE—Soluble Receptor for Advanced Glycation End-Products; CCL2—Chemokine (C-C motif) Ligand-2. Notes: Reference ranges are derived from previously collected healthy pediatric samples from UCK (*n* = 8) used for assay validation. Arrows (↑/↓) indicate direction of post-intervention change relative to baseline.

## Data Availability

The data presented in this study are available on request from the corresponding author.

## Supplementary Materials

The following supporting information can be downloaded at: https://www.mdpi.com/article/10.3390/jcm14238419/s1, CARE Checklist (2013) of information to include when writing a case report.
